# Antitumor Effect of Various Phytochemicals on Diverse Types of Thyroid Cancers

**DOI:** 10.3390/nu11010125

**Published:** 2019-01-09

**Authors:** Hye-Ji Shin, Kyung-A Hwang, Kyung-Chul Choi

**Affiliations:** Laboratory of Biochemistry and Immunology, College of Veterinary Medicine, Chungbuk National University, Cheongju, Chungbuk 28644, Korea; hzee1234@naver.com

**Keywords:** thyroid cancer, phytochemicals, reactive oxygen species, radio iodine therapy, apoptosis

## Abstract

Thyroid cancers developed from the tissues of the thyroid gland are classified into papillary (PTC), follicular (FTC), medullary (MTC), and anaplastic thyroid cancer (ATC). Although thyroid cancers have been generally known as mild forms of cancer, undifferentiated MTC and ATC have a more unfavorable prognosis than differentiated PTC and FTC because they are more aggressive and early metastatic. A variety of therapies such as surgery, radiotherapy, and chemotherapy have been currently used to treat thyroid cancer, but they still have limitations including drug resistance or unfavorable side effects. Phytochemicals are plant-derived chemicals having various physiological activities that are expected to be effective in cancer treatment. In this review, anticancer efficacy of phytochemicals, such as resveratrol, genistein, curcumin, and other substances in each type of thyroid cancer was introduced with their chemopreventive mechanisms. English articles related with thyroid cancer and anti-thyroid cancer of phytochemicals were searched from PubMed and Google Scholar. This article mainly focused on in vitro or animal studies on phytochemicals with anti-thyroid cancer activity. These various phytochemicals have been shown to induce apoptosis in all types of thyroid cancer cells, inhibit cell proliferation and invasion, and to be helpful in enhancing the effect of radioiodine therapy that is a typical therapy to thyroid cancer. These results suggest that thyroid cancer can be more effectively treated by the combinations of phytochemicals and the existing therapies or substances.

## 1. Introduction

The thyroid is a butterfly-shaped organ located in the middle of the human neck. It is an endocrine gland that generates and secretes hormones such as thyroxin and calcitonin; these hormones play an important role in metabolic regulation in the human body. Abnormal proliferation and the acquisition of metastatic potential of thyroid tissue cells form thyroid cancer. Globally, the number of people with thyroid cancer reached 3.2 million by 2017 [1]. Women are three times more likely to be affected with thyroid cancer than men and it is the fifth most common disease in total women cancers [2,3]. There are a number of environmental and genetic predisposing factors related with thyroid cancer, and it has been long known that thyroid cancer may occur by genetic causes, including multiple endocrine neoplasia type 2, or by radiation exposure of the thyroid gland [4,5]. An external radiotherapy in childhood has been accepted for greater frequency of thyroid cancer [6].

Although thyroid cancers have been generally known as mild forms of cancer, their mortality rate and severity vary according to its histopathologic types. Thyroid cancers are divided into differentiated thyroid cancer (DTC) and undifferentiated thyroid cancer. DTC includes papillary thyroid cancer (PTC) and follicular thyroid cancer (FTC). Undifferentiated thyroid cancers include medullary thyroid cancer (MTC) and anaplastic thyroid cancer (ATC). Differentiated types of thyroid cancers have a more favorable prognosis than undifferentiated MTC and ATC because poorly-differentiated cancers are generally more malignant and early metastatic than well-differentiated cancers [7]. PTC comprises 75% to 85% of cases of thyroid cancers, occurring often in females. Conversely, ATC, which is less than 5% of total thyroid cancer, has a low survival rate. ATC has a high mortality rate of 86% because no appropriate treatment is available [8]. MTC is frequently caused by rearranged during transfection (RET) gene mutations and is an uncommon cancer that arises from parafollicular cells that produce calcitonin [9]

Various therapies have been developed for thyroid cancers, i.e., surgery, radioiodine therapy, chemotherapy, and heat-therapy. In general, most of the thyroid cancers are treated surgically; the entire thyroid gland is removed. When cancer cells migrate to the lymph node around the neck, the lymph node should also be removed. Thyroid surgery is very simple and quick to treat, but the removal of the thyroid gland needs to take a thyroid hormone replacement pill every day. In addition, the metastatic thyroid cancer requires additional therapy, including radioiodine and hormone therapy. Since iodines tend to be absorbed by thyroid tissue, radioactive iodine selectively migrates to the thyroid and destroy thyroid cells, including thyroid cancer cells, having little side effects on other organs. Additionally, radiotherapy can reduce the recurrence and death rate of PTC and FTC patients by ablating residual cancer tissue after surgery [10]. However, radioiodine therapy can cause swelling and pain in the neck, it is less effective in metastatic thyroid cancers like MTC and ATC or radioiodine resistant advanced types of cancer. In addition, chemotherapy has been widely used for the treatment of thyroid cancers, and various drugs including sorafenib and lenvatinib are approved for advanced chemotherapy [11]. Most drugs cause drug-resistance when used for a long time, leading to the increased rate of recurrence. Therefore, more reliable therapies and agents for thyroid cancer treatment still need to be introduced.

Phytochemicals are chemical substances that are naturally produced in plants. They are easily accessible through ingesting various plant-including foods. They have various physiological activities including anti-inflammation, anti-oxidation, and anti-cancer effects with fewer side effects. Specifically, the efficacies of phytochemicals to induce cell growth regulation, apoptosis, anti-oxidation, and anti-angiogenesis are associated with their anti-cancer effects in various cancers [12]. In this review, we discuss the anti-cancer effects of phytochemicals that turned up in the studies for each type of thyroid cancer and how they can be helpful in combination with other therapies.

## 2. Methods

The information in this review article was searched in PubMed and Google Scholar using keywords such as “thyroid cancer”, “phytochemicals”, “reactive oxygen species”, “radio iodine therapy”, and “apoptosis”. All mentioned articles were searched until August 2018. This article mainly focused on the findings or outcomes from in vitro or animal studies on anti-thyroid cancer activity of phytochemicals.

## 3. Anticancer Effects of Phytochemicals on Thyroid Cancers

### 3.1. Resveratrol

Resveratrol (3,5,4′-trihydroxy-trans-stilbene) is a natural stilbenoid that has two aromatic rings with methylene bridge. It is present in grapes, blueberries, raspberries, and red wines and in peanuts, pomegranates, and soy beans [13,14]. Resveratrol has been known to be a biologically-active substance that can treat the diseases including obesity, inflammation, diabetes, and cancers [15,16,17,18]. Resveratrol has been shown to effectively inhibit growth in many types of cancer including breast, gastric, lung, and thyroid cancer [19,20,21,22].

For the case of thyroid cancer, resveratrol was found to be helpful in radioiodine therapy. Radioactive iodine generates reactive oxygen species (ROS) by triggering radiolysis of water in thyroid tissues, which disrupts the antioxidant defense system and causes chromosomal breaks and DNA damage as sides effects [23,24]. However, co-treatment with resveratrol with radioactive iodine can protect normal cells from ROS-induced cytotoxicity because hydroxyl groups in the chemical structure of resveratrol reduce free radicals [25,26]. In addition to its ROS scavenging effect, resveratrol has anti-thyroid cancer activities by regulating thyroid cancer-specific signaling pathways. In ATC, resveratrol was found to enhance re-differentiation and inhibit growth of HTh7 and 8505C cell lines by activating the Notch1 signaling pathway, which accumulate thyroid-specific differentiation markers [27]. In human MTC (TT) cells, resveratrol induces apoptosis via the Notch2 signaling pathway and suppresses neuroendocrine markers that are correlated with poor prognosis [28]. Resveratrol also induces p53 through Ras-MAPK kinase-MAPK signal transduction and increases proapoptotic agents such as c-fos, c-jun, and p21 in PTC cell lines (BHP 2–7 and BHP 18–21) and FTC cell lines (FTC 236 and FTC 238) [29].

Retinoic acid (RA) is known to be used for re-differentiation therapy in thyroid cancers [30]. However, RA-resistance thyroid cancers have difficulties in this treatment. To solve this problem, resveratrol was treated with RA in THR-11T cell line, which is a RA-resistant ATC cell line. It could improve the RA sensitivity by increasing the expression of RA tumor suppressor receptor, retinoic acid receptor (RAR)-β, and decreasing the expression of RA tumor promoter receptor, PPAR-β/α [31]. Therefore, the treatment with resveratrol enhances RA sensitivity and allows re-differentiation of thyroid cancer to delay tumorigenesis and malignant transformation of RA-resistant ATC cells.

In carcinogen-induced Sprague-Dawley rat models of thyroid cancer, intragastric and intraperitoneal injection of resveratrol significantly reduced the frequency and severity of thyroid cancer-related lesions such as hyperplasia and adenomas [32]. In the thyroid tissue administered with resveratrol, the expression of IL-6 and cyclooxygenase-2 was down-regulated, however, NF-κB/p65 nuclear translocation was reduced and IkB*α* expression was elevated.

The more cancer stem cells (CSCs) exist in cancers, the greater aggressiveness and drug resistance increase. When treated with resveratrol and valproic acid which is used to treat epilepsy, they induced CSC differentiation and enhanced the expression of thyroid differentiation markers, which resulted in reduced cell proliferation and invasiveness and increased apoptosis of thyroid CSCs [33].

These results imply that resveratrol facilitates to overcome ROS-induced cytotoxicity in radioiodine therapy and RA resistance in re-differentiation therapy and to induce thyroid tumor prevention via various pathways.

### 3.2. Isoflavone

Isoflavones are abundant in soybeans [34], and also found in legumes, lupine, fava bean, kudzu, and coffee [35,36]. Isoflavones, such as genistein and daidzein, are known to act as tyrosine kinase inhibitors and have biological functions, such as inhibition of topoisomerase and glycine receptors [37,38,39].

In some thyroid cancers, epidermal growth factor (EGF) induces the proliferation, migration, and invasion of differentiated thyroid cancer cells, for instance, of FTC and PTC in vitro and in vivo [40]. EGF is activated by the binding of transforming growth factor alpha (TGF-α) to EGF receptor and phosphorylates tyrosine kinase [41]. When tyrosine kinase is phosphorylated, it can regulate various protein functions, such as enzyme activity, interactions between molecules, and subcellular localizations, or also contribute to the progression of cancers [42]. This response can be blocked by antagonists to tyrosine kinase and EGF receptor. Genistein, an antagonist of tyrosine kinase, can neutralize EGF and TGF-α, which can inhibit the growth and invasion of the FTC cell line (FTC133) [43]. Gain-of-function mutations of the RET proto-oncogene leads to increase tyrosine kinase activity in MTC [44]. Additionally, in this case, genistein can inhibit cell proliferation and RET signaling of the MTC cell line (TT cells, RETc634 mutant) as a tyrosine kinase inhibitor (TKI) [45,46].

Genistein can be used in thyroid cancer therapy in combination with other substances. Photodynamic therapy (PDT) used to treat cancer uses photosensitizers (PS) or light sensitive drugs. PS can photo-excite at specific wavelengths in the PS spectrum to localize to malignant tissue and produce cytotoxic singlet oxygen which damages cells and induces cell death [47]. In the recent study for the anti-thyroid cancer effect of the combination of genistein and photofrin, one of the photosensitizers, genistein enhanced photofrin mediated PDT against SNU-80 ATC cells by increasing ROS level and modifying the expression of apoptosis related proteins [48]. Studies have also been carried out to co-treat thymoquinone (TQ), which is abundant in *Nigella sativa,* with genistein to thyroid cancer cells. TQ has antioxidant, anti-cancer, anti-angiogenic, and anti-proliferative effects in various cancers. The combination of TQ and genistein decreased telomerase activity, angiogenesis, and cell survival, and increased apoptosis in thyroid cancer cells more effectively in ATC cells than in FTC cells [49,50,51]. These combination therapies not only improve the anti-cancer effect but also reduce side effects by reducing the amount of drug used in the treatment of ATC [52].

The synthetic derivative of daidzein, N-t-Boc-7-(O)-carboxymethyl daidzein (cD-tboc), is used in the treatment of various thyroid cancer cells. cD-tboc can induce cell apoptosis and not necrosis of thyroid cancer cells like FTC (MRO 87-1 and WRO), PTC (NPA), and ATC (ARO 81-1) [53,54]. The apoptotic effect also found in MTC (TT cell), cD-tboc induces cell apoptosis and necrosis [55].

Genistein, a tyrosine kinase inhibitor that neutralizes the effects of growth factors, such as EGF and TGF-α, can inhibit cell growth and invasion of FTC of MTC cells. In addition, when treated with other anticancer substances, such as PS or TQ, genistein can reinforce their effects to inhibit progression of thyroid cancer. Additionally, cD-tboc, which is a synthetic derivative of daidzein, can induce apoptosis and inhibit cell viability in all types of thyroid cancer cells.

### 3.3. Curcumin

One of the ginger family, turmeric (*Curcuma longa*), is a plant commonly used for curry powder in south and southeast tropical Asia. The rhizome of turmeric is the most useful part for cookery and medicinal purpose [56]. In turmeric, the most active component is curcumin, ((1E, 6E)-1,7-Bis(4-hydroxy-3-methoxyphenyl)hepta-1,6-diene-3,5-dione), which has a variety of biological functions, such as antibacterial, anti-inflammatory, antimicrobial, and anti-cancer effects [57].

In solid tumor, hypoxia is a common condition in which tumor cells are deprived of oxygen due to their rapid proliferation, leading to increased production of hypoxia-inducible factor-1 (HIF-1) [58]. HIF-1 is composed of HIF-1α and HIF-1β, and acts as a master oxygen homeostasis regulator. During hypoxia, HIF-1 binds to the hypoxia response element (HRE) and initiates gene expression. As a result, the expression of matrix metallopeptidase 9 (MMP-9) and E-cadherin, which are characteristic proteins of tumor metastasis and invasion, are regulated [59]. In KI PTC cells in hypoxia, curcumin reduced the binding capacity of HIF-1α to HRE and decreased the expression of HIF-1α responsive genes. As a result, curcumin inhibited migration and invasion by promoting expression of E-cadherin and suppressing activation of MMP-9 [60,61]. In a short period of curcumin treatment, ROS was accumulated and induced apoptosis of PTC cells. ROS accumulation also contributed to the collapse of MMP and to increase the intracellular Ca^2+^ influx amount, leading to apoptosis and inhibition of migration and invasion of K1 PTC cells [62].

The PI3K/Akt pathway is known to increase the expression of MMPs, which facilitate tumor progression including growth, invasion, and metastasis [63]. Curcumin inhibited invasion and migration of FTC 133 cells via down-regulation of PI3K/Akt signaling pathway and inhibition of MMP-1, MMP-7 and cyclooxygenase-2 (COX-2) [64]. Additionally, since overexpressed COX-2 is associated with tumor angiogenesis [65], COX-2 inhibition by curcumin can suppress tumor angiogenesis. Treatment of curcumin with sorafenib, a kinase inhibitor drug approved for the treatment of certain types of cancer, effectively inhibited cell proliferation via PI3K/Akt and extracellular signal-regulated kinase (ERK) pathway in 133 FTC cells [66]. Another pathway of metastasis is TGF-β1/smad2/3 signaling pathway in which TGF-β1 phosphorylates smad2 and smad3 and activates MMPs through the smad2/3 pathway to induce metastasis in thyroid cancer. Curcumin-inhibited TGF-β1-induced epithelial-mesenchymal transition (EMT) through down-regulation of the smad2/3 signaling pathway in PTC BCPAP cells [67].

Activation of nuclear factor-κB (NF-κB) often occurs in tumor cells and contributes to aggressive tumor growth and resistance to chemotherapy and radiotherapy [68]. Down-regulation of NF-κB can also be detected in various thyroid cancer cell lines, BHT-101 (ATC), FTC-133 (FTC) and TPC-1 (PTC), treated with curcumin [69]. This down-regulation of NF-κB can increase the therapeutic efficacy by lowering the threshold for cell apoptosis of docetaxel, which is known as an anti-cancer agent in ATC [70]. Curcumin also promoted the therapeutic effect of radioactive iodine and can be expected to provide effective treatment in radiotherapy [71].

Curcumin induces cell cycle arrest in a variety of cells including thyroid cancer, leading to apoptosis. Curcumin caused the activation of the cell apoptosis cascade through G2/M phase cell cycle arrest in PTC BCPAP cells [72]. Additionally, curcumin inhibited cell cycle progression of TPC1 PTC cells by downregulating cyclin D1 [73].

From these studies, it was found that curcumin inhibited EMT, migration, and invasion of thyroid cancer cells by regulating HIF-1α, PI3K/Akt, and TGF-β pathways, and NF-κB. In addition, curcumin can increase therapeutic efficacy of drugs for the treatment of thyroid cancer including sorafenib and docetaxel.

### 3.4. Miscellaneous Phytochemicals

Anti-cancer effects on thyroid cancers have been also demonstrated in other various phytochemicals, i.e., myricetin, quercetin, epigallocatechin-3-gallate (EGCG), and apigenin. Myricetin is found in nuts or berries, quercetin in fruits or vegetables, and apigenin (4′,5,7-trihydroxyflavone) in parsley, celery, celeriac, and chamomile tea, and EGCG is the main catechin of green tea [14].

In the radioiodine therapy for thyroid cancers, myricetin increased the intake and retention of iodide in the FTC 133 cell line via Na^+^/I^−^ symporter (NIS) [74]. The increased retention of radioiodine by myricetin is a striking contrast from the cases of apigenin, luteolin, and kaempferol that decrease retention of radioiodine by increasing the efflux of iodide [75]. Thus, myricetin can enhance the effectiveness of radioiodine therapy.

Quercetin is known as an inhibitor of heat shock protein (Hsp), which can be used in cancer therapy. Treatment with quercetin can induce apoptosis by down-regulating Hsp90, which inhibits the activation of chymotrypsin-like proteasome in PTC B-CPAP cell line [76,77]. In heat therapy, hyperthermia is known to damage cancer cells due to high temperature [78]. In contrast, Hsp70 protects cancer cells from heat-induced apoptosis and inhibits killing effect on cancer cells. Here, quercetin is used to enhance hyperthermia by down-regulating Hsp70 as an inhibitor [79]. In addition, quercetin, like myricetin, increased NIS, which is a differentiation marker, in FTC-133 (FTC), NPA (PTC), FRO (ATC) cell lines, and decreased the expression of CD97, a de-differentiation marker in TPC-1 (PTC), FTC-133, NPA, FRO, and ARO (ATC) cell lines [80]. Moreover, EGCG inhibits the proliferation of cancer cells through epidermal growth factor receptor (EGFR) and ERK pathways and downregulates cell cycle-associated protein, resulting in apoptosis in ATC cell line, ARO [81].

Apigenin with combination of an Akt inhibitor also can increase the iodine flux rate for enhancing effectiveness of radioiodine therapy in FTC. For this effect apigenin needs the p38 MAPK activation, which can increase radioiodine uptake [82]. Furthermore, apigenin induces apoptosis via c-Myc, which is known to modulate p53 in ATC FRO cell line [83,84]. Unlike other phytochemicals, apigenin stimulates ROS production, which leads to DNA damage and autophagic cell death in PTC BCPAP cell line [85].

## 4. Conclusions

In general, the treatment for thyroid cancers is considered to be relatively easy because the rate and frequency that metastasis occurs are slower and lower, respectively. However, it is difficult to treat poorly-differentiated types of thyroid cancer, such as MTC and ATC, which contribute to high mortality rates. At present, surgery, chemotherapy, and radioiodine therapy, etc., have been adopted for thyroid cancer treatment, however, there are unexpected problems, such as drug resistance or side effects related with killing normal cells, which should be solved.

Phytochemicals are plant-derived chemicals and have diverse physiological activities. In particular, they have received much attention because they have a therapeutic effect on various cancers with few side effects. The antitumor effects of many kinds of phytochemicals on thyroid cancer have been also studied in vitro and in vivo. Resveratrol, genistein, curcumin, and other phytochemicals have been shown to induce apoptosis in all types of thyroid cancer, to be effective for the combined treatment with radioiodine therapy, and for poorly-differentiated types of thyroid cancer through diverse mechanisms as summarized in Table 1.

However, there are still problems awaiting solutions such as enhancement of bioavailability of phytochemicals. Even if polyphenols are abundantly ingested from the diet, they do not necessarily reach target tissues in the form of active metabolites at high concentrations. There exist several factors that affect bioavailability of phytochemicals, such as water solubility, elimination half-life, and relative urinary excretion [87]. Fortunately, it has been known that isoflavones have a good bioavailability as the most well-absorbed polyphenols [87]. Resveratrol, however, has been known to have low bioavailability, and methods using bio enhancers and nano-formulations have been recommended to increase its solubility and tissue absorption [88]. The efforts to improve the bioavailability of phytochemicals should continue in order to increase their applicability as biomedicine. In addition, comparing with experimental studies, clinical or epidemiological evidence for anti-thyroid cancer effects of phytochemicals is sparse. Therefore, more etiological or clinical studies are needed to clarify a role of phytochemicals in thyroid cancer prevention and to increase their applications for treatment of thyroid cancers.

## Figures and Tables

**Table 1:** Anti-thyroid cancer mechanisms of phytochemicals.

Phytochemicals	Anti-Cancer Effect	References
Resveratrol	• Improve RA sensitivity • Enhance the efficacy of radiotherapy • Induce apoptosis or re-differentiation • CSC differentiation	[31] [25] [27,28,29,32] [33]
Isoflavones	• Inhibit proliferation • Decrease invasion • Induce apoptosis • Enhance the efficacy of photodynamic therapy	[46,54,55] [43] [52,53,55] [48]
Curcumin	• Produce ROS in short time • Suppress ROS production • Decrease EMT • Inhibit invasion • Enhance the efficacy of radiotherapy • Induce apoptosis • Inhibit proliferation	[62] [60] [61,67] [61,64] [69,71] [66,72] [73]
miscellaneous	Myricetin • Enhance the efficacy of radiotherapy Quercetin • Induce re-differentiation • Induce apoptosis • Enhance the efficacy of hyperthermia Epigallocatechin-3-gallate • Inhibit proliferation • Decrease EMT Apigenin • Enhance the efficacy of radiotherapy • Induce apoptosis • Product ROS to induce autophagic cell death	[75] [80] [76,77] [79] [81,86] [86] [82] [84] [85]

## Abbreviations

DTC	differentiated thyroid cancer
PTC	papillary thyroid cancer
FTC	follicular thyroid cancer
MTC	medullary thyroid cancer
ATC	anaplastic thyroid cancer
RET	rearranged during transfection
ROS	reactive oxygen species
RA	retinoic acid
RAR	retinoic acid receptor
CSCs	cancer stem cells
EGF	epidermal growth factor
TGF-α	transforming growth factor alpha
TKI	tyrosine kinase inhibitor
PDT	photodynamic therapy
PS	photosensitizers
HIF-1	hypoxia inducible factor-1
HRE	hypoxia response element
MMP-9	matrix metallopeptidase 9
COX-2	cyclooxygenase-2
ERK	extracellular signal-regulated kinase
EMT	epithelial-mesenchymal transition
NF-κB	nuclear factor-κB
EGCG	epigallocatechin-3-gallate
NIS	Na+/I-symporter
Hsp	heat shock protein
EGFR	epidermal growth factor receptor
